# Sex-specific effects of neonatal progestin receptor antagonism on juvenile social play behavior in rats

**DOI:** 10.1186/s12993-021-00183-z

**Published:** 2021-11-05

**Authors:** R. M. Forbes-Lorman

**Affiliations:** Department of Biology, Ripon College, 300 W Seward St., Ripon, WI 54971 USA

**Keywords:** Rat, Sex differences, Progesterone, Progestin receptor (PR), Social play behavior, Social discrimination

## Abstract

Developing mammals are exposed to progesterone through several sources; however, the role of progesterone in early development is not well understood. Males express more progestin receptors (PRs) than females within several brain regions during early postnatal life, suggesting that PRs may be important for the organization of the sex differences in the brain and behavior. Indeed, previous studies showed cognitive impairments in male rats treated neonatally with a PR antagonist. In the present study, we examined the role of PRs in organizing juvenile behaviors. Social play behavior and social discrimination were examined in juvenile male and female rats that had been treated with CDB, a PR antagonist, during the first week of postnatal life. Interestingly, neonatal PR antagonism altered different juvenile behaviors in males and females. A transient disruption in PR signaling during development had no effect on social discrimination but increased play initiation and pins in females. These data suggest that PRs play an important role in the organization of sex differences in some social behaviors.

## Introduction

While it is clear that testosterone and its metabolites play an important role in the organization of the male brain and behavior through their actions on androgen receptors and estrogen receptors [1], less is known about the role of progesterone acting upon progestin receptors (PRs) in the developing brain.

Developing mammals are exposed to progesterone, both from fetal and maternal sources (reviewed in [2, 3]). Indeed, male and female rodents have approximately equivalent levels of circulating progesterone during development [4, 5]. Furthermore, progesterone administration is also commonly used as a contraceptive in lactating women and during pregnancy for prevention of premature birth and (reviewed in [6]).

Males express PRs as early as embryonic day 20 in many brain areas [7] and express more PRs than females within several hypothalamic regions on postnatal day (PN)1 but not PN10 [8, 9]. This sex difference in PRs in the developing brain suggests that PRs are important for the organization of the sex differences in the brain. Indeed, blocking PRs using RU-486 during development increases male sex behavior and the expression of ARs in several regions of the adult male brain [10], although another study using a different PR antagonist, ZK 137616, found no effect on male mouse sex behavior [11]. Neonatal PR antagonism using RU-486 also disrupts cognitive ability in adult male rats [12]. It should be noted that RU-498 also binds glucocorticoid receptors, making the role of PRs during development even less clear. CDB-4124 is a PR antagonist with a low binding affinity for glucocorticoid receptors [13] that has been shown to affect forced swim immobility in adult mice [14].

Several studies have also examined the role of progestins in the organization of social behaviors. For example, neonatal progesterone administration increased play in both sexes [15], while progestin antiserum decreased play only in females [16]. Progesterone has also been demonstrated to play a role in adult male social behaviors. For example, progesterone administration has been found to disrupt social recognition in adult males [17], while a more recent study showed the opposite [18].

In the present study, we examined the organizational role of PRs on juvenile social discrimination and juvenile social play behavior by treating male and female rats with CDB, a specific PR antagonist, during the first week of postnatal life.

## Methods and materials

### Subjects and treatment

Sprague–Dawley rats supplied by Charles River Labs were bred in our animal facility. Animals were housed in standard lab cages with aspen shavings and no enrichment. Dams were checked daily to determine the day of birth and were allowed to deliver normally. Twenty-seven male and 25 female pups were pooled from five different litters and randomly assigned to each treatment group (13 CDB-treated females, 12 oil-treated females, 13 CDB-treated males, and 14 oil-treated males). Each litter contained animals of both sexes and treatment groups and a maximum of three animals from a single litter were assigned to each treatment group. Pups were foot-marked with India ink and treated subcutaneously with the 75 g/0.01 mL/g body of the PR antagonist CDB-4124 or vehicle on PN0 (day of birth), PN2 and PN4. The vehicle was composed of 0.2% benzyl alcohol and 0.6% benzyl benzoate in sesame oil. This treatment regimen is similar to what has been previously used for RU-486 [10]; however, we chose to reduce the number of administrations in order to minimize the injections. We have found that the sesame oil does not clear within a day, so treatment was likely continuous from ~ PN0-PN6. The weight of the pups ranged from 5 to 12 g over the three days of treatment. CDB-4124 was used because it has a low binding affinity for glucocorticoid receptors [13]. The dose of CDB-4124 is within the range of what has previously had an effect on forced swim immobility in adult mice [14].

**Fig. 1:**
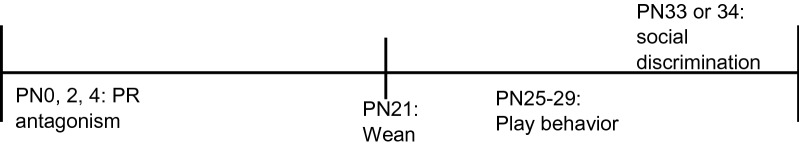
Experimental overview. Male and female rats were injected with the PR antagonist CDB or vehicle control on PN0, 2, and 4 then weaned into mixed-sex cages of 5–6 on PN21. Play behavior was observed in the home cage from PN25-29 and social discrimination was measured on PN33 or PN34

All pups remained with dams until weaning at PN21. On PN21, pups were separated into seven cages of six animals and two cages of five animals. In order to minimize litter effects, each cage contained 1–2 animals from each treatment group (i.e., CDB-treated females, CDB-treated males, vehicle-treated females, vehicle-treated males) and approximately half females and half males. An overview timeline of the experiment is shown in Fig. 1. The rats were housed under a 12:12 light/dark cycle with food and water available ad libitum*.* This research was approved by the University of Wisconsin Institutional Animal Care and Use Committee.

### Behavioral testing

Behavioral tests were performed under dim red light approximately 1–2 h after the beginning of the dark phase of the light cycle. Each behavior was recorded and then analyzed by a trained technician blind to all treatments using The Observer^®^ (Noldus Information Technologies) or Stopwatch + (Center for Behavioral Neuroscience, Atlanta, GA).

### Social play behavior

The play behavior paradigm and scoring criteria were adapted from previous studies [19, 20] which both use the focal observation method to capture a “snapshot” of the play occurring in each home cage. On PN25-29, play behavior was digitally recorded in two 4-min sessions per day in the home cage covered with a clear plastic lid. One play session was 2 h after the beginning of the dark period and one play session was 4 h after the beginning of the dark session. Therefore, we recorded 8-min of play occurring in each home cage every day for five days, for 40 min total. There were 5–6 animals in each cage, randomly numbered and coded by tail marks. An observer blind to the treatment groups scored the recordings for the following individual behaviors: pin, pounce, bite, and chase. The frequency of each play behavior was calculated by summing each animal’s play behaviors over the entire observation time. While there are different methods used to analyze play behavior, frequency of play behaviors is the most common [21]. The animals from one cage were not used in the final analysis because this cage contained animals of two different ages and social play changes with age [22, 23]. Animal numbers for play behavior were therefore 12 CDB-treated females, 11 oil-treated females, 12 CDB-treated males, and 11 oil-treated males).

### Social discrimination

On PN33 or PN34, rats were tested for social discrimination. Although it is typical to isolate adult animals for 1–10 days prior to testing [17, 24, 25], we isolated the juvenile animals for only 4 h in this study, as social isolation is considered a severe stressor for juvenile animals [26]. In trial 1, an age and sex-matched juvenile stimulus rat was placed in the home cage of the experimental animal and the experimental animal was allowed to freely investigate for five minutes. After five minutes, the stimulus juvenile was removed and the experimental animal was alone in its cage for 30 min. After this 30-min intertrial interval, the stimulus juvenile from trial 1 and a stimulus novel juvenile were placed in the experimental animal's cage, and the experimental animal was again free to investigate for five minutes. The juvenile stimulus rats were distinguishable by unique tail marks drawn with permanent marker. Investigation of the stimulus juveniles was scored two ways: (1) body investigations, which included direct contact between the nose of the experimental animal and the body of the stimulus juvenile; and (2) anogenital investigations, which included direct contact between the nose of the experimental animal and the anogenital region of the stimulus juvenile. Percent novel investigation was calculated by dividing the time spent investigating the novel animal divided by the time spent investigating either animal, multiplied by 100. Percentages greater than 50% indicate discrimination and larger scores indicate better discrimination. Animal numbers for social discrimination were 13 CDB-treated females, 12 oil-treated females, 13 CDB-treated males, and 14 oil-treated males.

### Statistical analyses

All statistical comparisons were carried out using SPSS v. 28 (IMB). Statistical comparisons were carried out using a two-way ANOVA and simple main effects were conducted to examine pairwise comparisons of the effect of treatment within each sex. Partial eta squared (Ŋ^2^) was used to estimate effect size, with Ŋ^2^ = 0.06 indicating a medium effect size and Ŋ^2^ = 0.14 indicating a large effect size [27].

## Results

### Social play behavior

**Fig. 2:**
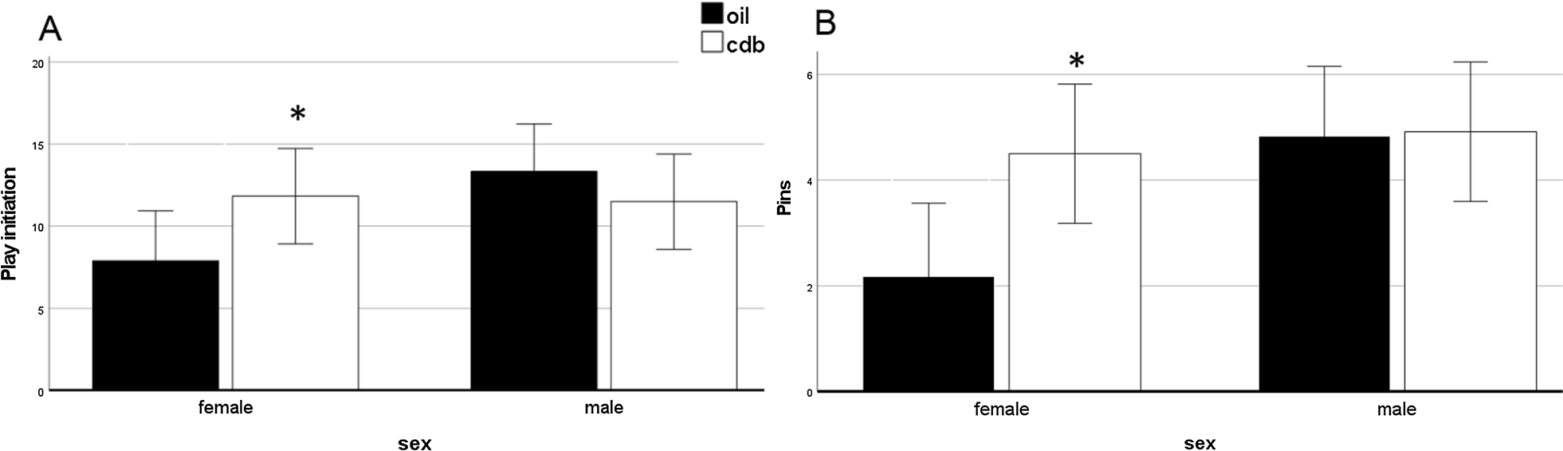
Social play in juvenile rats injected with CDB or control vehicle on PN0, 2, and 4. Each bar represents the mean total number of instances of play. Error bars represent 2× SEM. **A** The interaction between sex and CDB treatment on play initiation approached significance (*p* = 0.05, Ŋ^2^ = 0.08). Simple main effects indicate that the effect of CDB in females approached significance (*p = 0.06), while there was no effect of CDB in males (*p* = 0.37). **B** There was a main effect of sex (*p* = 0.03, Ŋ^2^ = 0.11), while the main effect of treatment (*p* = 0.08, Ŋ^2^ = 0.07) and interaction between sex and treatment (*p* = 0.09, Ŋ^2^ = 0.06) approached significance (*p* = 0.05, Ŋ^2^ = 0.08). Simple main effects indicate CDB-treated females pinned more than control females (*p = 0.02), while there was no effect of CDB in males (*p* = 0.93)

There was no main effect of sex [F(1,47) = 3.1, *p* = 0.09, Ŋ^2^ = 0.07] or treatment F(1,47) = 0.52, *p* = 0.48, Ŋ^2^ = 0.01 on the initiation of play behavior; however, the interaction between sex approached significance [*F*(1,47) = 3.9, *p* = 0.05, Ŋ^2^ = 0.08, Fig. 2A]. Simple main effects indicate that the effect of CDB in females approached significance (*p* = 0.06), while there was no effect of CDB in males (*p* = 0.37). There was a main effect of sex [*F*(1,47) = 5.4; *p* = 0.03, Ŋ^2^ = 0.11] on pins, while the main effect of treatment [*F*(1,47) = 3.3; *p* = 0.08, Ŋ^2^ = 0.07] and interaction between sex and treatment [*F*(1,47) = 2.8, *p* = 0.09, Ŋ^2^ = 0.06] approached significance (Fig. 2B). Simple main effects indicate CDB-treated females pinned more than control females (*p* = 0.02), while there was no effect of CDB in males (*p* = 0.93). There were no main effects or interactions on pounces or chases (Table 1). Bites were observed only several times total, so these were not analyzed independently.

**Table 1:** Total frequency of play behaviors from PN25-29, recorded for 8 min per day (40 min total) in the home cage

Play parameter	Control males	Control females	CDB males	CDB females
Pounces	7.6 ± 1.0	5.5 ± 0.9	6.2 ± 2	6.5 ± 0.9
Pins	4.8 ± 0.7	2.2 ± 0.5^a^	4.9 ± 0.6	4.5 ± 0.9
Chases	0.9 ± 0.3	0.3 ± 0.2	0.4 ± 0.2	0.7 ± 0.2
Initiation	13.3 ± 1.7	7.9 ± 1.5^a^	11.5 ± 1.2	11.8 ± 1.6

There were no effects of sex or treatment on chases or pounces

^a^Control females exhibited fewer pins and total initiation behaviors compared to CDB-treated females, as shown in Fig. 2

Frequency of play parameters (mean ± standard error).

### Social discrimination

**Fig. 3:**
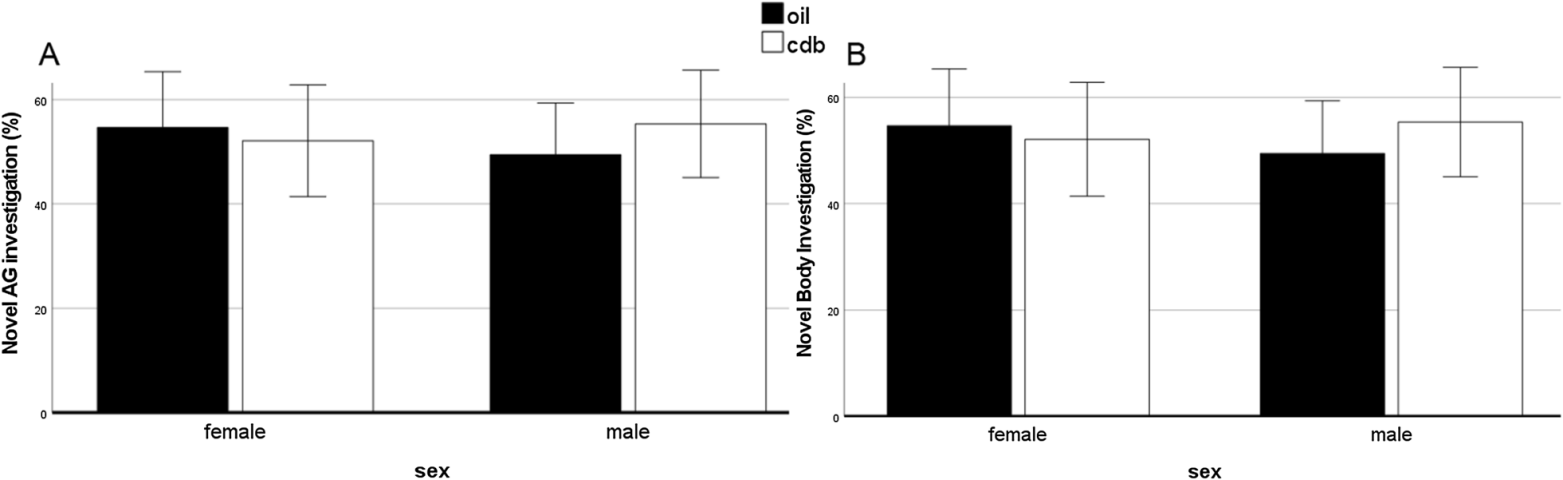
There were no effects of sex or treatment on social discrimination in juvenile rats injected with CDB or control vehicle on PN0, 2, and 4. Error bars represent 2× SEM. Each bar represents the mean percent of time that each group performed anogenital (AG) investigations (**A**) or body investigations (**B**) with the novel animal, with percentages greater than 50% indicating discrimination

There was no effect of sex [*F*(1,50) = 0.47; *p* = 0.50] or treatment [*F*(1,50) = 2.5; *p* = 0.12] on percent novel anogenital investigations (Fig. 3A) and no effect of sex [*F*(1,50) = 0.36; *p* = 0.42] or treatment [*F*(1,50) = 1.1; *p* = 0.75] on percent novel body investigations (Fig. 3B).

## Discussion

In the present study, males initiated play more than females and exhibited more pins, which is consistent with much of the previous literature using focal observation [19, 21, 28]. Additionally, the interaction between sex and treatment approached significance. Specifically, it appears that neonatal PR antagonism increased play initiation and pins in females, with no effect in males. Interestingly, there were no sex or treatment effects on chases or pounces, suggesting that the increase in play initiation in CDB-treated females is driven primarily by increases in pins. The reason for this is unclear, but it is particularly interesting given that pinning may be indicative of dominance [21, 29]. These results are also consistent with a recent study demonstrating a sex difference in pins and total play, but not chases, in both mixed-sex and same-sex pairs [30]. On the other hand, there was no effect on social discrimination following neonatal PR antagonism.

As antagonizing PRs during early life increases pins and play initiation in females, PR signaling may play a role in organizing female social play behavior. Even though levels of PRs in the developing female brain are lower than levels in the developing male brain, their action may be important for preventing masculinization in females. That is, during the first week of postnatal life, PRs appear to be important for establishing female-typical levels of play. Although previous data have demonstrated that PRs regulate social behavior in adults [17, 25], the present study is the first to show effects on juvenile social play behavior following a transient neonatal manipulation of PRs. Effect sizes for all reported statistics are in the medium-large range.

The mechanism for the increase in play in females following neonatal manipulation of PRs is unclear, as little is known about the role of PRs in the developing postnatal female brain. Manipulation of PRs may affect a variety of signaling molecules, such as arginine vasopressin, opioids, endocannabinoids, dopamine, norepinephrine, serotonin, and GABA, which are all involved in social play behavior [31–33]. Prior studies have found an increase in arginine vasopressin expression in the lateral habenula of PR knockout mice [34], but it is unclear if these effects are due to altered PRs in development or adulthood. Further studies are necessary to elucidate the relationship between PRs and juvenile social play.

In the present study, there was no effect on social discrimination following neonatal PR antagonism. Although previous data have demonstrated that PRs regulate social discrimination in adult male rats [17, 25], the present data suggest that PRs do not play an organizational role in this behavior during the first week of postnatal life. It should be noted that social discrimination is a complex behavior, and several other signaling molecules have been shown to play a role in social discrimination, including oxytocin [35–37] and arginine vasopressin [24, 38–40].

To my knowledge, the present study is the first to examine the specific role of PRs during early development on juvenile social behaviors. In females, there was an increase in play initiation, while there was no effect in males. Future studies should examine the role of neonatal PRs on the neurobiology of the brain and behavior in both juvenile and adult animals.

## Data Availability

The datasets used and/or analysed during the current study are available from the corresponding author on reasonable request.

## Abbreviations

PR: Progestin receptor
PN: Postnatal
